# Supplementary material for: A systematic review of fluralaner as a treatment for ectoparasitic infections in mammalian species
Source: PeerJ. 2025 Mar 12;13:e18882. doi: 10.7717/peerj.18882 (PMC11910153; doi:10.7717/peerj.18882)
Supplement: Supplemental Information 2 [file peerj-13-18882-s002.docx]

Intended audience:

Scientists and veterinarians
